# Genome-wide Association Study for Starch Pasting Properties in Chinese Spring Wheat

**DOI:** 10.3389/fgene.2022.830644

**Published:** 2022-03-25

**Authors:** Yousheng Tian, Wei Sang, Pengpeng Liu, Jindong Liu, Jishan Xiang, Fengjuan Cui, Hongjun Xu, Xinnian Han, Yingbin Nie, Dezhen Kong, Weihua Li, Peiyuan Mu

**Affiliations:** ^1^ The Key Laboratory of the Oasis Ecological Agriculture, College of Agriculture, Shihezi University, Shihezi, China; ^2^ Department of Administrative Management, Xinjiang Academy of Agri-reclamation Sciences, Shihezi, China; ^3^ Institute of Crop Science, Xinjiang Academy of Agri-reclamation Sciences/Key Lab of Xinjiang Production and Construction Corps for Cereal Quality Research and Genetic Improvement, Shihezi, China; ^4^ Institute of Crop Sciences, Chinese Academy of Agricultural Sciences, Beijing, China

**Keywords:** candidate genes, GWAS, haplotype analysis, RVA parameters, *Triticum aestivum*

## Abstract

In order to understand the genetic basis of starch pasting viscosity characteristics of Chinese spring wheat, we assessed the genetic variation of RVA parameters determined by the Rapid Visco Analyser in a panel of 192 Chinese spring wheat accessions grown in Er’shi, Shihezi and Zhaosu during 2012 and 2013 cropping seasons. A genome-wide association study with 47,362 single nucleotide polymorphism (SNP) markers was conducted to detect marker-trait associations using mixed linear model. Phenotypic variations of RVA parameters ranged from 1.6 to 30.7% and broad-sense heritabilities ranged from 0.62 to 0.91. Forty-one SNP markers at 25 loci were significantly associated with seven RVA traits in at least two environments; among these, 20 SNPs were located in coding sequences (CDS) of 18 annotation genes, which can lead to discovering novel genes underpinning starch gelatinization in spring wheat. Haplotype analysis revealed one block for breakdown (BD) on chromosome 3B and two blocks for pasting temperature (T) on chromosome 7B. Cultivars with superior haplotypes at these loci showed better starch pasting viscosity than the average of all cultivars surveyed. The identified loci and associated markers provide valuable sources for future functional characterization and genetic improvement of starch quality in wheat.

## Introduction

Wheat (*Triticum aestivum* L.) is one of the most important staple food crops worldwide. With the improvement of living standards, people pay more attention to the quality of end-use products of wheat. Improvement of quality traits has become a major objective in wheat breeding (Kong et al., 2013). The gelatinization characteristic of wheat flour is a main index to evaluate the processing quality of food products (Crosbie, 1991; Panozzo et al., 1993; Liu et al., 2003; Kaur et al., 2016; Amiri et al., 2018; Moiraghi et al., 2019). Rapid Visco Analyser (RVA) profile has proven useful in wheat breeding programs to assess the eating and cooking quality of wheat (Konik et al., 1992; He et al., 2003; He et al., 2004; Zhang et al., 2004; Zhang et al., 2005; León et al., 2006). Starch pasting viscosity is controlled by multiple genes and often influenced by environments (Zhang et al., 2009), the traditional methods for assessing RVA parameters are laborious and need expensive equipment. The use of molecular markers for an indirect marker-assisted selection (MAS) is effective in selection for quality traits in breeding process. Therefore, it is important to study the genetic basis of starch gelatinization for wheat quality improvement using MAS.

Previous studies have been heading for localizing genes and QTL for starch gelatinization characteristics to expedite MAS in wheat breeding (Udall et al., 1999; Araki et al., 2000; Deng et al., 2014). However, QTL identified by bi-parent populations cannot explain the variation of starch gelatinization characteristics in complex genetic population due to relatively simple genetic background and lower allele variability. The genome-wide association study (GWAS) can identify genomic regions associated with variations in a given trait by combining phenotypic with genotypic data (AL-Maskri et al., 2012). Compared with conventional bi-parental QTL mapping, GWAS has the advantage of surveying a larger range of allelic variations and avoiding a time-consuming process for establishing a customized mapping population (Tadesse et al., 2015). Because of having more genetic diversity and historical recombination of alleles among associated panels, GWAS can get more accurate results (Muhu-Din Ahmed et al., 2020). With development of sequencing technology, high-density wheat SNP arrays have been developed, which combined with GWAS were widely used to identify genetic loci for important traits in hexaploid wheat (Sukumaran et al., 2015; Sun et al., 2017; Rimbert et al., 2018; Zhang et al., 2018; Yan et al., 2019; Lv et al., 2020; Shi et al., 2020), especially for quality-related traits, such as grain protein content, wet gluten content, grain starch content, SDS-sedimentation volume, dough rheological properties, and so on (Muqaddasi et al., 2020; Yang et al., 2020; Muhu-Din Ahmed et al., 2020), but none for starch pasting properties.

Herein, we performed a GWAS to identify genetic loci for RVA parameters using the wheat 90K SNP array and multi-environment field data in a panel of 192 Chinese spring wheat genotypes. Markers significantly associated with RVA parameters and candidate genes were identified. The results of this study can enhance our understanding of the genetic basis of wheat starch gelatinization and provide valuable information for MAS in wheat breeding.

## Materials and Methods

### Plant Materials and Field Trials

A set of 192 genetically diverse spring wheat accessions, representing cultivars and breeding lines from different provinces of China, was grown at Er’shi, Shihezi and Zhaosu in Xinjiang province in randomized complete blocks with three replications during 2012 and 2013 cropping seasons (hereafter referred as 2012_ES, 2012_SHZ, 2012_ZS, 2013_ES, 2013_SHZ and 2013_ZS, respectively). Each genotype was sown in ten rows, with a row length of 3 m, a row-to-row distance of 25 cm and plant-to-plant distance of 10 cm. There were differences in climate and soil conditions among Er’shi, Shihezi and Zhaosu, and different temperatures between the years 2012 and 2013. Planting and harvest dates and trial management varied according to the recommendations of each location. The accessions were harvested at maturity and cleaned prior to quality test.

### Milling

Flour milling was performed in a mill (MLU202, Wuxi, China) to flour extraction rates of around 65%. Prior to milling, the hard, medium hard (mixtures of hard and soft wheat) and soft wheats were tempered overnight to moisture contents of around 16, 15, and 14%, respectively.

### Measurements of RVA Parameters

Pasting properties of flour were determined with a Rapid Visco Analyser (RVA-Techmaster, Newport Scientific, Australia). The 3 g flour was suspended in 25 ml of distilled water before the solution was placed inside RVA instrument. The programs of temperature in the following order: held at 50°C for 60 s, heated from 50 to 95°C at a rate of 1°C/5 s and held at 95°C for 150 s, then cooled to 50°C at a rate of 1°C/5 s and held at 50°C for 120 s. RVA parameters including peak viscosity (PV), trough viscosity (TV), breakdown (BD), final viscosity (FV), setback (SB), peak time (PT) and pasting temperature (T) were determined.

### Genotyping

Fresh leaf samples were collected from 10-day old seedlings and sent to the CapitalBio Technology company^1^ in Beijing for genotyping with the high-density illumina wheat 90K SNP array. After excluding the low-quality SNP markers with minor allele frequency (MAF) ≤0.02 and missing data ≥10%, 47,362 SNPs were used for GWAS. All SNP markers were anchored on the wheat genome (IWGSC RefSeq v1.0) using BLASTN by 50 bp SNP flanking sequences on both sides of the SNP.

### Structure Analysis

The Bayesian clustering technique was used with 3400 SNP markers to classify groups of genotypically same individuals using the statistical software STRUCTURE v.2.3.4 (Pritchard et al., 2000). Burn-in iterations of 10^4^ cycles were used, followed by a simulation runs of 10^5^ cycles with an admixture model. The K values of 1–10 and 3 independent runs were selected to attain reliable results. Web-based analysis “Structure Harvester v0.6.93”^2^ was applied to obtain maximum value or peak of “K” for validation to understand the STRUCTURE v.2.3.4 results using ad-hoc techniques (Earl and VonHoldt, 2011). ΔK was plotted against the number of sub-group K following Evanno et al. (2005).

A principal component analysis (PCA) using filtered SNPs was performed with the TASSEL v.5.2.43 and the first three PCA values were plotted in three dimensions.

### Genome-Wide Association Study

Phenotypic data in different environments and best linear unbiased prediction (BLUP) values were analyzed, respectively, for association analysis to identify the marker-trait associations (MTAs) employing mixed linear model (MLM) in TASSEL v.5.2.43 (Bradbury et al., 2007). The MLM option requires population structure (Q-matrix) and kinship matrix (K-matrix) as covariates for GWAS to avoid false positives. The Q-matrix was generated through STRUCTURE v.2.3.4, whereas the K-matrix was generated by TASSEL v.5.2.43. The Bonferroni multiple testing correction was used to identify significant markers. Significant SNPs associated with RVA traits were claimed when the significance test reached *p* < 4.18E-4 (20/47,362 = 0.000418). Linkage disequilibrium (LD) was calculated by TASSEL 5.0 with the markers whose positions were known. The LD decay plot was generated by intra-chromosomal *r* ^2^ and base pair distance using R package ggplot2.

### Mapping SNPs and Prediction of Candidate Genes

The Chinese spring reference genome (IWGSC RefSeq v1.0) and gene annotations in GFF3 format were retrieved from the Ensemble database release 44^3^. The SNP marker sequences were mapped to the wheat genome using BLASTN program with a stringent E-value of 0.0001. For each SNP only the best scoring hit was retained. Each aligned genomic position was annotated into 5′-UTR, 3′-UTR, CDS, intron and intergenic region according to the genomic regions provided in the GFF3 file. The intergenic region was the genomic region with no annotated genes. The protein functions of candidate genes were predicted in the Uniprot Protein database^4^.

### Haplotype Analysis

For the genomic regions harboring SNPs with -log10 (*p*) above the threshold, and the phenotypic values of accessions with different alleles reached significant level (*p* < 0.05) in multi-environment, haplotype analysis was carried out by Haploview version 4.2 software (Barrett et al., 2005), and candidate loci were determined by testing the significant differences on phenotypes among major haplotypes through analysis of variance (ANOVA).

### Statistical Analysis

ANOVA and correlation analysis were carried out using SPSS version 22.0. The coefficient of variation (CV) was calculated by dividing the standard deviation by the average of trait values. The BLUP values of all traits over 2 years across environments were calculated by the R package Lme4 (Bates et al., 2015). Broad-sense heritabilities (*h* ^*2*^) of RVA traits were calculated as *h* ^*2*^ = *σ* ^*2*^_*g*_/(*σ* ^2^_*g*_ + *σ* ^2^_*gl*_/*l* +*σ* ^2^_*gs*_/*s + σ* ^2^_*gls*_/*ls*), where *σ* ^*2*^_*g*_ *, σ* ^2^_*gl*_, *σ* ^2^_*gs*_, *σ* ^2^_*gls*_ variances for genotypic, genotype by location interaction, genotype by season interaction and genotype by location by season interaction, respectively, whereas *l* and *s* were the numbers of locations and seasons, respectively. SNP density plots, Manhattan and quantile-quantile (Q-Q) plots were generated in R package CMplot while histograms were performed in Origin 8.0.

## Results

### Phenotypic Distributions and Correlations of RVA Parameters

The variation coefficients of PV, TV, BD, FV, SB, PT and T were in the ranges of 8.4–13.6%, 7.0–12.9%, 20.4–30.7%, 5.4–12.6%, 7.6–12.9%, 1.6–3.0%, 1.9–13.6%, respectively, across environments, and *h* ^*2*^ estimates were 0.91, 0.77, 0.86, 0.89, 0.82, 0.62 and 0.85, respectively (Supplementary Table S1). Except the T, the frequency distributions of BLUP values of RVA parameters were nearly symmetrically distributed (Supplementary Figure S1). RVA parameters exhibited wide phenotypic variations and high broad-sense heritabilities, which were imperative for an efficient GWAS.

Based on the BLUP values across six environments, PV was significantly and positively correlated with TV (r = 0.68**), BD (r = 0.81**) and FV (r = 0.63**); TV was significantly and positively correlated with FV (r = 0.94**), SB (r = 0.72**) and PT (r = 0.63**); FV was significantly and positively correlated with SB (r = 0.89**) and PT (r = 0.48**); BD was significantly and negatively correlated with PT (r = −0.43**, Table 1).

**TABLE 1:** Pearson’s correlation coefficients (r) between RVA parameters.

Trait	PV	TV	BD	FV	SB	PT
TV	0.68^a^					
BD	0.81^a^	0.13				
FV	0.63^a^	0.94^a^	0.10			
SB	0.36^a^	0.72^a^	−0.09	0.89^a^		
PT	0.03	0.63^a^	−0.43^a^	0.48^a^	0.31^a^	
T	−0.24^a^	−0.14	−0.22^a^	−0.13	−0.04	0.01

PV, peak viscosity; TV, trough viscosity; BD, breakdown; FV, final viscosity; SB, setback; PT, peak time; T, pasting temperature.

a Indicates significance levels at *p* < 0.01.

### Genome-wide Associations

**FIGURE 1:**
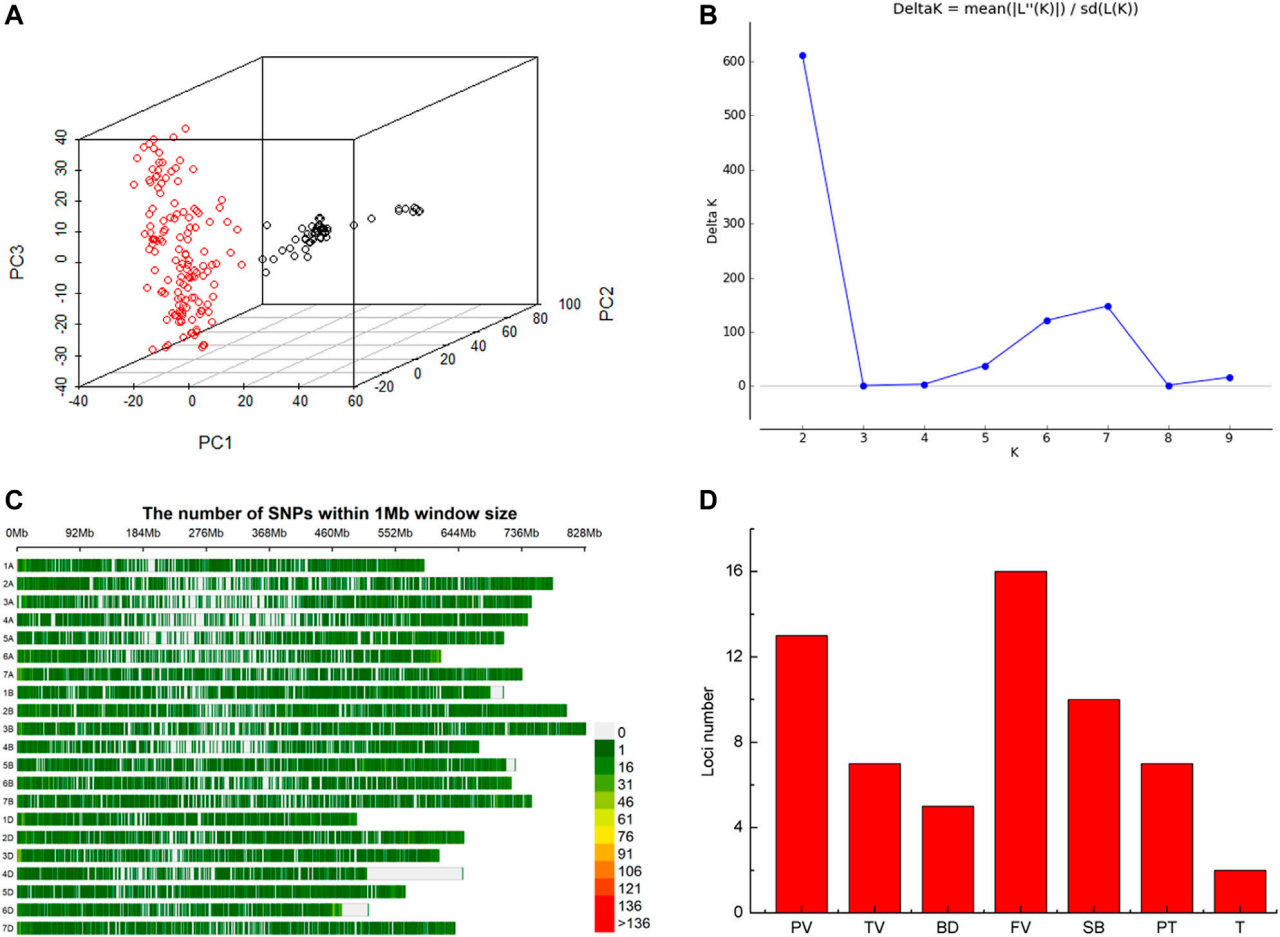
Structure of the association panel of 192 Chinese spring wheat varieties for genome-wide association study (GWAS), distribution of SNPs and significant loci revealed by GWAS. **(A)** Structure of association panel. **(B)** Broken line of delta K. **(C)** Distribution of SNPs on chromosomes. **(D)** Histogram plot of loci number significantly associated with RVA parameters *via* GWAS based on BLUP values.

**FIGURE 2:**
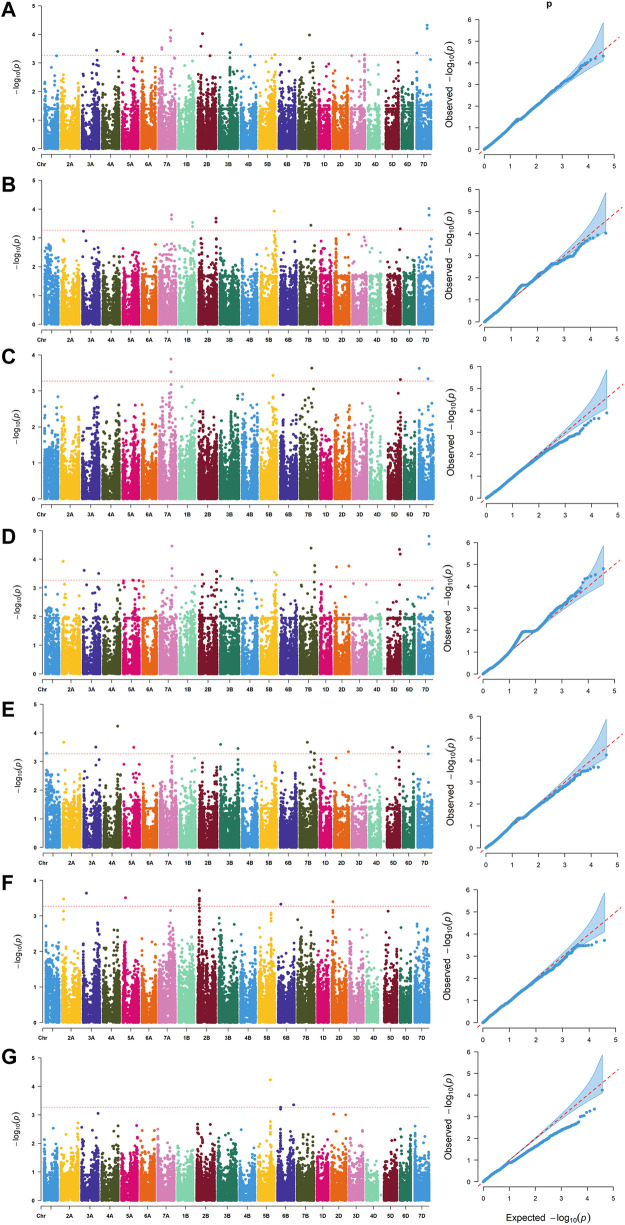
Manhattan and quantile-quantile **(Q–Q)** plots for RVA parameters identified by genome-wide association study based on BLUP values. **(A)** peak viscosity (PV). **(B)** trough viscosity (TV). **(C)** breakdown (BD). **(D)** final viscosity (FV). **(E)** setback (SB). **(F)** peak time (PT). **(G)** pasting temperature (T).

Based on whole-genome genotyping data, PCA showed that the association panel could be divided into two subgroups (Figure 1A), the same with the results calculated by the STRUCTURE software with 3,400 markers, in which the peak of the broken line graph was observed at k = 2 (Figure 1B), indicating the association panel can be divided into two sub-populations. After filtering low-quality markers, 47,362 SNPs were used for GWAS analysis with BLUP values and individual data in six environments. The number of SNPs within 1 Mb window size in chromosomes indicated that these SNPs were almost evenly distributed across the whole genome (Figure 1C). To avoid multiple significances within individual LD blocks, the support interval was determined when the decay distance of LD reached *r* ^2^ = 0.2, and LD was estimated to decay at about 7 Mb for whole genome (Supplementary Figure S2). Manhattan and Q-Q plots based on BLUP values identified 92 significant SNPs at 51 loci (Figure 2; Supplementary Table S2), of which 13, 7, 5, 16, 10, 7 and 2 were significantly associated with PV, TV, BD, FV, SB, PT and T, respectively (Figure 1D). Phenotypic variations explained by these SNPs ranged from 6.9 to 11.1% for PV, 6.6–8.4% for TV, 7.2–8.8% for BD, 5.9–11.9% for FV, 5.2–7.3% for SB, 6.7–7.7% for PT, and 6.8–9.1% for T (Supplementary Table S2).

### Significant and Stable SNPs Associated With RVA Parameters and Candidate Genes

Forty-one SNPs at 25 loci were stably detected in at least two environments (BLUP is considered as an environment, Supplementary Table S3), and four SNPs (*GENE-4428_113, BobWhite_c13098_670, IAAV4275, Kukri_rep_c69088_774*) on chromosomes 7A (1), 7B (1) and 7D (2) at 534, 489 and 463 Mb, respectively, were significantly associated with both PV and TV, while one SNP (*Excalibur_c9183_1397*) on chromosome 7D at 13 Mb was significantly associated with both PV and BD. Thirty-four SNP markers associated with RVA parameters were mapped in the annotated genes; 20 SNPs among these were located in the CDS of 18 annotation genes, which were considered as candidate genes (Supplementary Table S4).

### Haplotype Analysis of Genetic Loci Related to RVA Parameters

To test the effect of different genotypes on RVA parameters, stable SNPs were selected to group the populations according to their genotypes, and *t*-test was used to test the significance of genotypic effects on the traits. Three SNPs (*BS00023017_51, Excalibur_c10124_361, Kukri_c4560_110*) revealed significant differences (*p* < 0.05) of the traits between two alleles in at least four environments (Supplementary Table S5), indicating that these loci had a great influence on phenotypic variation.

**FIGURE 3:**
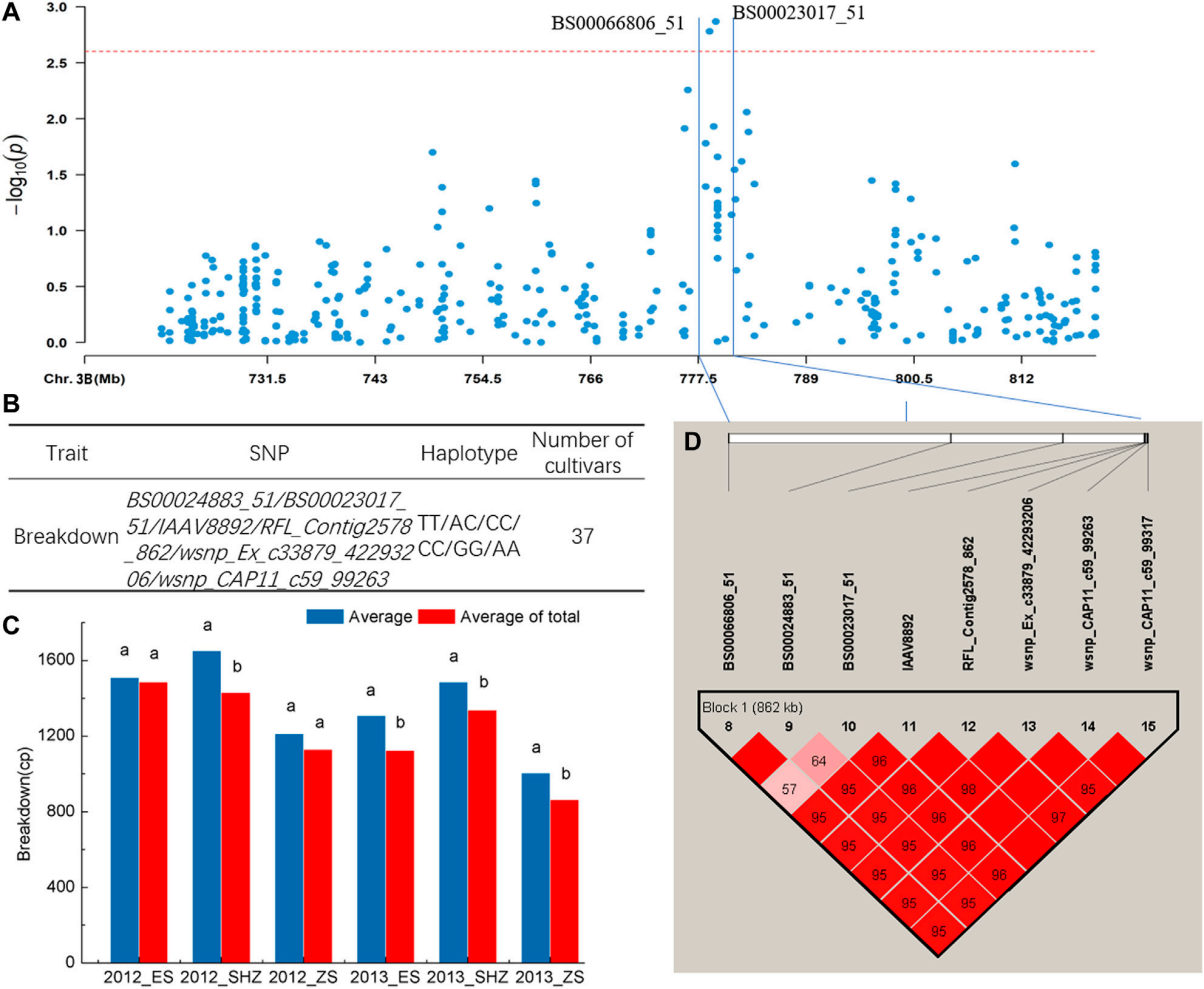
Haplotype analysis. **(A)** Local Manhattan plot. **(B)** Haplotypes with different SNP alleles in the block. **(C)** Phenotypic effects of haplotypes in different environments, Different lowercase letters indicate significant differences at *p* < 0.05. **(D)** Haplotype analysis of multi-environment significant SNPs associated with breakdown on chromosome 3B, the color represents the linkage between SNPs, and the deeper color means the higher linkage between SNPs.

The haplotype analysis for regions harboring the SNP marker (*BS00023017_51*) associated with BD showed that the 776–782 Mb interval on chromosome 3B had an 862-kb block, and six SNPs including *BS00023017_51* were clustered in the block (Figures 3A,D). Comparison of BD values indicated that 37 cultivars with the TT/AC/CC/CC/GG/AA (*BS00024883_51/BS00023017_51/IAAV8892/RFL_Contig2578_862/wsnp_Ex_c33879_42293206/wsnp_CAP11_c59_99263*) haplotypes showed significantly higher BD (*p* < 0.05) than the average of all cultivars surveyed in four environments (2012_SHZ, 2013_ES, 2013_SHZ, 2013_ZS, Figures 3B,C). Haplotype analysis was also conducted for region harboring the SNPs (*Excalibur_c10124_361, Kukri_c4560_110*) associated with T, and two blocks in 442–454 Mb interval on chromosome 7B were detected. Two cultivars with CC/AA/CC (*CAP12_c8025_110/IAAV 2037/Excalibur_c49622_60*), 17 cultivars with AA/TT/GG/GG (*wsnp_Ex_c64815_63464750/RAC875_c4438_419/IAAV3414/TA001679-0356*) haplotypes showed higher T than the average of all cultivars surveyed, exhibiting significant differences in some locations (*p < 0.05*, Supplementary Table S6, Supplementary Figure S3).

## Discussion

### Phenotypic Variations and Heritabilities in RVA Parameters

Identification of genetic loci controlling wheat quality parameters is useful in breeding programs (Matus and Hayes, 2002; Mourad et al., 2020). In the association panel evaluated in this study, we observed significant phenotypic variations among accessions in RVA parameters (Supplementary Table S1). Earlier researches were in line with current results that starch pasting properties exhibited large variations (He et al., 2006; Ram and Sharma, 2013). Successive phenotypic distributions indicated polygenic inheritance of RVA parameters (Supplementary Figure S1). Heritability estimation provides information about the extent of a particular genetic character to be transmitted to offspring (Muhu-Din Ahmed et al., 2020). In this experiment, RVA parameters had high heritabilities (Supplementary Table S1), similar with a previous study, which also reported a high heritability in RVA parameters (Rahim et al., 2020). Our results demonstrated that this panel of wheat varieties had high levels of intra-species genetic flow, which made it suitable for the genetic study of RVA parameters by GWAS.

### GWAS for RVA Parameters

Marker-trait association study established the relationship between specific phenotypic and genetic variability within a genome, which ultimately detected loci underpinning corresponding traits. The ability to capture significant associations between polymorphic loci and phenotypic variance depend on the extent of LD along the genome (Pritchard and Przeworski, 2001; Remington et al., 2001). LD decay was about 7 Mb in our association panel in the present study, which consistent with previous studies (Appels et al., 2018; Yang et al., 2020), but inconsistent with most studies (Sukumaran et al., 2015; Jamil et al., 2019; Roncallo et al., 2019; Muhammad et al., 2020; Hu et al., 2021; Sharma, 2021), because of the differences in the type of markers used for genotyping and the sample size variation in the different studies (Chao et al., 2010). In the past, many studies for protein quality in wheat have been reported (Kristensen et al., 2019; Yang et al., 2020), whereas, few reports are about QTL for RVA parameters. In this study, we identified SNP markers associated with RVA traits using MLM. Besides the BLUP value, GWAS was also conducted with data of individual environments as a reference for locating SNPs that were relatively stable across different experimental environments. We highlighted those SNPs detected in at least two environments as stable SNPs and 41 significant MTAs were identified (Supplementary Table S3).

### Loci for RVA Parameters

Significant and stable MTAs for PV were mainly clustered to chromosomes 2B, 3A, 3B, 4A, 4B, 5A, 7A, 7B and 7D (Supplementary Table S3); several were consistent with previous studies (Udall et al., 1999; Sun et al., 2008; Zhang et al., 2009; Deng et al., 2014). *Wx-B1* gene (*TRAESCS4A02G418200*, chr4A, 688,097,145–688,100,962 bp) encodes granule-bound starch synthase, generating a higher PV and BD (Briney et al., 1998; Araki et al., 2000; Batey et al., 2002; Miura et al., 2002; Ram and Sharma, 2013). Some studies found the QTL for starch pasting properties near *Wx-B1* on chromosome 4A (Batey et al., 2002; Mccartney et al., 2006). In the present study, we also identified a SNP marker (*BobWhite_c17731_56*, Chr.4A: 689,849,795 bp, Supplementary Table S3) associated with PV, its physical position was very close to *Wx-B1* gene. *TRAESCS7D02G365900* on chromosome 7D at 473,617,760 to 473,624,545 bp, encodes phosphorylase which probably contributes to starch synthesis and degradation (Tetlow et al., 2004; Tickle et al., 2009; Mishra et al., 2016). In the current study, we found two SNP markers (*IAAV4275* and *Kukri_rep_c69088_774*) around there.

Our results corroborated other QTL studies in wheat, where QTL associated with BD was detected on chromosome 3B (Supplementary Table S3). However, we did not find QTL for BD on chromosomes 1A, 1B, 3A, 4A, 4B, 6A and 6D (Zhang et al., 2009; Deng et al., 2014). We detected new QTL (*GENE-4993_69* on chromosome 7B, *Excalibur_c9183_1397* and *Tdurum_contig69003_459* on chromosome 7D, Supplementary Table S3) were not reported in previous studies. One stable SNP (*BS00067650_51*, Supplementary Table S3) associated with FV was detected at 526,464,187 bp on chromosome 5D in this study which was never reported before, while several FV QTL were identified on chromosomes 1A, 1B, 1D, 3A, 3D, 4A, 4B, 5B, 5D, 6B, 6D and 7A previously (Batey et al., 2002; Mccartney et al., 2006; Sun et al., 2008; Zhang et al., 2009; Deng et al., 2014). Using two DH populations, Batey et al. (2002) found QTL for FV near *Wx-B1* on chromosome 4A, where no locus associated with FV was detected in this research. Nine SNPs in seven loci on chromosomes 2B (2), 5B (1), 6B (1), 7B (2) and 6D (1) were significantly associated with T (Supplementary Table S3), whereas QTL for T were reported on chromosomes 2D, 3A, 3B, 4A, 4B, 5D, 6D in previous studies (Zhao et al., 2009; Deng et al., 2014). The MTA at *BS00022437_51* (chr.6B, 715,775,218 bp) significantly associated with T was very close to *TRAESCS6B02G418100* (chr.6B, 690,447,513–690,452,026 bp) which was the homolog of phosphorylase (PHO, Tetlow et al., 2004; Li et al., 2018).

We also detected eight stable SNPs at five loci for TV on chromosomes 7A, 2B, 5B, 7B and 7D, one SNP for SB on chromosome 5A, and one SNP for PT on chromosome 2A (Supplementary Table S3). Whereas, previous studies have reported more loci on other chromosomes (Sun et al., 2008; Deng et al., 2014), which were not detected in this study, because alleles at these loci might be fixed in this association panel, or rare alleles cannot be detected.

### Candidate Genes

The annotation conducted on 41 stable SNPs identified by GWAS showed that seven (17%) SNPs were mapped in intergenic regions, and 34 (83%) were mapped in genic regions (Supplementary Table S4). Among these, 20 SNPs were located in the CDS of 18 annotation genes which will result in discovering new genes controlling RVA parameters in wheat. Furthermore, *GENE-4428_113, BobWhite_c13098_670*, *IAAV4275* and *Kukri_rep_c69088_774* had effects on both PV and TV, and *Excalibur_c9183_1397* had effect on both PV and BD; these SNPs were all mapped in the CDS of annotation genes, which may be key candidate genes that participate in regulating wheat starch quality. *Excalibur_c9183_1397* was located in the CDS region of *TraesCS7D02G026700* encoding 1,3-beta-glucan synthase that is present mainly in the cell walls of starchy endosperm (Moravčíková et al., 2016). Other candidate genes that were not reported in previous research need to be paid more attention for study in the future.

### Haplotype Analysis

In the present study, SNPs significantly associated with RVA parameters were identified on almost all wheat chromosomes. To validate the effect of each SNP, estimations of SNP effects were used to predict the observed phenotypic performance (Supplementary Table S5). *BS00023017_51*, *Excalibur_c10124_361* and *Kukri_c4560_110* had significant (*p* < 0.05) effects on traits in at least four environments, and could be used as optimal loci in marker-assisted breeding and quality improvement. The haplotype analysis was conducted on these loci and three blocks were detected (Figure 3, Supplementary Figure S3; Supplementary Table S6). Cultivars with superior haplotypes showed relatively better phenotypes than the average of all cultivars surveyed. Therefore, these loci can be considered to improve cultivars for starch quality.

## Conclusion

It is not feasible that phenotypic selection for improving wheat starch quality at the early stages in wheat breeding. Even at later stages in the breeding process, starch quality was also impacted greatly by environments. Therefore, genetic selection through MAS is a desirable way to improve wheat starch quality in wheat breeding programs. Based on dense SNPs across the whole genome, the GWAS has become a common approach to uncover genetic components of agronomic traits, which provides us with insightful information into genetic architecture of complex traits. In this study, GWAS analysis were performed for RVA parameters with 47,362 SNPs in 192 Chinese spring wheat accessions among six environments. Forty-one SNPs at 25 loci were stably detected in at least two environments, of which 20 SNPs were located in the CDS of 18 annotation genes. Haplotype analysis for regions harboring the SNPs (*BS00023017_51, Excalibur_c10124_361, Kukri_c4560_110*) revealed one block for BD on chromosome 3B and two blocks for T on chromosome 7B, cultivars with superior haplotypes at these loci showed better starch pasting viscosity than the average of all cultivars surveyed. Validation studies for SNPs in the candidate genes and detected loci will be conducted in the future by designing KASP assays, which can be further used for marker-assisted breeding for improvement of grain starch quality in wheat.

## Data Availability

The data presented in the study are deposited in Figshare DOI: 10.6084/m9.figshare.19297601.

## Footnotes

1
https://www.capitalbiotech.com/.

2
http://taylor0.biology.ucla.edu/structureHarvester/.

3
ftp://ftp.ensemblgenomes.org/pub/plants/release-44.

4
https://www.uniprot.org/.
