# Supplementary material for: Genome-wide Association Study for Starch Pasting Properties in Chinese Spring Wheat
Source: Front Genet. 2022 Mar 25;13:830644. doi: 10.3389/fgene.2022.830644 (PMC8990798; doi:10.3389/fgene.2022.830644)
Supplement: Supplementary file 1 [file DataSheet1.docx]

Table S1 Phenotypic variations and heritabilities of RVA parameters

| Trait | Environment | Minimum | Maximum | Mean | SD | CV (%) | *h^2^* |
| --- | --- | --- | --- | --- | --- | --- | --- |
| PV  (cp) | 2012_ES | 2881 | 4478 | 3810.64 | 331.58 | 8.7 | 0.91 |
|  | 2013_ES | 2402 | 4365 | 3347.14 | 387.96 | 11.6 |  |
|  | 2012_SHZ | 1918 | 5482 | 3630.82 | 492.14 | 13.6 |  |
|  | 2013_SHZ | 3097 | 4549 | 3705.99 | 311.13 | 8.4 |  |
|  | 2012_ZS | 2678 | 4342 | 3488.76 | 292.00 | 8.4 |  |
|  | 2013_ZS | 2390 | 4153 | 3097.41 | 374.40 | 12.1 |  |
| TV  (cp) | 2012_ES | 1911 | 2901 | 2325.94 | 204.96 | 8.8 | 0.77 |
|  | 2013_ES | 1433 | 2954 | 2222.31 | 254.11 | 11.4 |  |
|  | 2012_SHZ | 1175 | 2725 | 2187.94 | 282.40 | 12.9 |  |
|  | 2013_SHZ | 3097 | 4549 | 2356.09 | 186.92 | 7.9 |  |
|  | 2012_ZS | 2012 | 2830 | 2359.95 | 164.77 | 7.0 |  |
|  | 2013_ZS | 1786 | 3023 | 2232.45 | 227.96 | 10.2 |  |
| BD  (cp) | 2012_ES | 745 | 2247 | 1484.71 | 316.24 | 21.3 | 0.86 |
|  | 2013_ES | 414 | 1846 | 1123.45 | 299.46 | 26.7 |  |
|  | 2012_SHZ | 386 | 2195 | 1430.16 | 329.00 | 23.0 |  |
|  | 2013_SHZ | 812 | 2142 | 1337.71 | 273.46 | 20.4 |  |
|  | 2012_ZS | 571 | 1695 | 1128.81 | 233.38 | 20.7 |  |
|  | 2013_ZS | 382 | 1638 | 863.72 | 265.12 | 30.7 |  |
| FV  (cp) | 2012_ES | 3323 | 4810 | 3993.30 | 289.91 | 7.3 | 0.89 |
|  | 2013_ES | 2772 | 4607 | 3814.15 | 334.27 | 8.8 |  |
|  | 2012_SHZ | 2202 | 5450 | 3829.52 | 481.22 | 12.6 |  |
|  | 2013_SHZ | 3266 | 4871 | 4026.06 | 272.85 | 6.8 |  |
|  | 2012_ZS | 3387 | 4379 | 3831.63 | 208.33 | 5.4 |  |
|  | 2013_ZS | 3204 | 4805 | 3719.73 | 296.59 | 8.0 |  |
| SB  (cp) | 2012_ES | 1304 | 2108 | 1663.06 | 150.85 | 9.1 | 0.82 |
|  | 2013_ES | 1298 | 1935 | 1593.39 | 136.61 | 8.6 |  |
|  | 2012_SHZ | 1027 | 2194 | 1634.47 | 210.25 | 12.9 |  |
|  | 2013_SHZ | 1305 | 1984 | 1670.46 | 142.01 | 8.5 |  |
|  | 2012_ZS | 1257 | 1803 | 1482.01 | 112.59 | 7.6 |  |
|  | 2013_ZS | 1250 | 1877 | 1487.19 | 131.64 | 8.9 |  |
| PT  (min) | 2012_ES | 5.67 | 6.73 | 6.22 | 0.19 | 3.0 | 0.62 |
|  | 2013_ES | 5.93 | 6.60 | 6.30 | 0.15 | 2.4 |  |
|  | 2012_SHZ | 5.67 | 6.60 | 6.19 | 0.18 | 2.9 |  |
|  | 2013_SHZ | 5.93 | 6.60 | 6.32 | 0.13 | 2.1 |  |
|  | 2012_ZS | 6.27 | 6.80 | 6.49 | 0.11 | 1.6 |  |
|  | 2013_ZS | 6.13 | 6.80 | 6.50 | 0.15 | 2.3 |  |
| T  (℃) | 2012_ES | 64.35 | 71.05 | 66.94 | 1.25 | 1.9 | 0.85 |
|  | 2013_ES | 63.65 | 87.20 | 71.12 | 8.47 | 11.9 |  |
|  | 2012_SHZ | 66.05 | 89.65 | 71.34 | 6.37 | 8.9 |  |
|  | 2013_SHZ | 65.20 | 86.45 | 69.85 | 6.30 | 9.0 |  |
|  | 2012_ZS | 63.50 | 88.10 | 70.59 | 8.90 | 12.6 |  |
|  | 2013_ZS | 64.30 | 88.85 | 72.06 | 9.80 | 13.6 |  |

PV, peak viscosity; TV, trough viscosity; BD, breakdown; FV, final viscosity; SB, setback; PT, peak time; T, pasting temperature. 2012_ES, 2012_SHZ, 2012_ZS, 2013_ES, 2013_SHZ and 2013_ZS represent 2012 and 2013 cropping seasons in Er’shi (ES), Shihezi (SHZ) and Zhaosu (ZS), respectively. SD, SD standard deviation. CV, coefficient of variations. *h^2^*, broad-sense heritability.

Table S2 Significant SNPs associated with RVA traits by genome-wide association study based on BLUP values

| Trait | Marker | Chromosome | Position (bp) | *P-*value | *R^2^*(%) |
| --- | --- | --- | --- | --- | --- |
| PV | *Tdurum_contig56373_348* | 3A | 644622716 | 3.60E-04 | 8.8 |
|  | *BobWhite_c17731_56* | 4A | 689849795 | 3.95E-04 | 9.0 |
|  | *RAC875_c55661_677* | 5A | 52409349 | 4.93E-04 | 8.4 |
|  | *BS00108779_51* | 7A | 139098843 | 2.91E-04 | 9.5 |
|  | *BS00091101_51* | 7A | 142648203 | 3.35E-04 | 9.6 |
|  | *wsnp_Ex_c11636_18742884* | 7A | 512762486 | 1.28E-04 | 8.1 |
|  | *GENE-4428_113* | 7A | 534326357 | 7.16E-05 | 9.7 |
|  | *Ku_c104966_604* | 7A | 534332644 | 1.35E-04 | 8.1 |
|  | *IAAV447* | 7A | 536516307 | 1.71E-04 | 7.8 |
|  | *wsnp_CAP12_rep_c4678_2134259* | 2B | 146634773 | 2.62E-04 | 8.6 |
|  | *BS00078506_51* | 2B | 210842786 | 9.46E-05 | 11.1 |
|  | *Ku_c23179_1250* | 3B | 487540433 | 4.37E-04 | 6.9 |
|  | *Tdurum_contig46867_799* | 4B | 7038188 | 2.29E-04 | 8.3 |
|  | *Ku_c2735_2959* | 5B | 695992955 | 5.11E-04 | 8.5 |
|  | *BobWhite_c13098_670* | 7B | 489827215 | 1.06E-04 | 8.4 |
|  | *RAC875_c61950_1644* | 3D | 604355667 | 5.17E-04 | 6.9 |
|  | *Excalibur_c9183_1397* | 7D | 13011013 | 4.51E-04 | 7.1 |
|  | *IAAV4275* | 7D | 463039931 | 4.84E-05 | 10.1 |
|  | *Kukri_rep_c69088_774* | 7D | 463039931 | 6.27E-05 | 9.9 |
| TV | *GENE-4428_113* | 7A | 534326357 | 1.59E-04 | 7.6 |
|  | *Kukri_rep_c69478_1058* | 7A | 536509639 | 2.21E-04 | 7.2 |
|  | *BS00066855_51* | 1B | 588487021 | 4.03E-04 | 6.6 |
|  | *BS00073257_51* | 1B | 589489117 | 2.91E-04 | 6.8 |
|  | *Kukri_c55909_1109* | 2B | 770681399 | 2.07E-04 | 7.5 |
|  | *Kukri_c11040_787* | 2B | 770684873 | 2.79E-04 | 7.2 |
|  | *Tdurum_contig70554_1004* | 5B | 604140767 | 1.17E-04 | 7.8 |
|  | *BobWhite_c13098_670* | 7B | 489827215 | 3.64E-04 | 6.6 |
|  | *GENE-3006_294* | 5D | 558441139 | 4.83E-04 | 8.4 |
|  | *IAAV4275* | 7D | 463039931 | 9.53E-05 | 8.1 |
|  | *Kukri_rep_c69088_774* | 7D | 463039931 | 1.61E-04 | 7.6 |
| BD | *wsnp_be488670A_Ta_2_1* | 7A | 512761112 | 2.96E-04 | 7.3 |
|  | *wsnp_Ex_c11636_18742884* | 7A | 512762486 | 1.30E-04 | 8.1 |
|  | *GENE-3383_710* | 5B | 550393681 | 3.73E-04 | 8.8 |
|  | *GENE-4403_405* | 7B | 521572294 | 2.32E-04 | 7.5 |
|  | *BS00009821_51* | 5D | 550509823 | 4.82E-04 | 8.8 |
|  | *Excalibur_c9183_1397* | 7D | 13011013 | 2.35E-04 | 8.1 |
|  | *GENE-4400_154* | 7D | 396985525 | 4.61E-04 | 7.2 |
| FV | *Excalibur_c54892_188* | 2A | 41985239 | 1.18E-04 | 8.1 |
|  | *Ku_c18096_552* | 3A | 45598422 | 2.45E-04 | 8.8 |
|  | *BS00081610_51* | 3A | 685781388 | 3.13E-04 | 8.0 |
|  | *GENE-4428_113* | 7A | 534326357 | 3.47E-05 | 9.1 |
|  | *Ku_c104966_604* | 7A | 534332644 | 2.09E-04 | 6.7 |
|  | *IAAV447* | 7A | 536516307 | 3.81E-04 | 6.1 |
|  | *wsnp_CAP12_rep_c4678_2134259* | 2B | 146634773 | 3.42E-04 | 7.3 |
|  | *Kukri_c55909_1109* | 2B | 770681399 | 2.63E-04 | 7.3 |
|  | *wsnp_CAP11_c1820_985143* | 2B | 782534075 | 2.69E-04 | 6.8 |
|  | *RAC875_rep_c71149_148* | 2B | 782537838 | 4.95E-04 | 5.9 |
|  | *RAC875_rep_c111384_441* | 2B | 782688172 | 2.63E-04 | 6.5 |
|  | *RAC875_rep_c69171_241* | 3B | 3603408 | 3.90E-04 | 8.0 |
|  | *BS00000498_51* | 3B | 526124209 | 4.81E-04 | 7.5 |
|  | *RFL_Contig2772_1693* | 5B | 605480942 | 2.85E-04 | 8.4 |
|  | *Ku_c2735_2959* | 5B | 695992955 | 3.46E-04 | 8.0 |
|  | *BobWhite_c13098_670* | 7B | 489827215 | 4.10E-05 | 8.3 |
|  | *BobWhite_c8890_279* | 7B | 650130699 | 1.66E-04 | 8.6 |
|  | *wsnp_Ex_c22955_32173776* | 7B | 650130699 | 2.78E-04 | 8.1 |
|  | *Excalibur_c5278_1075* | 2D | 88610742 | 1.85E-04 | 8.5 |
|  | *RAC875_c1858_2693* | 2D | 627926191 | 1.71E-04 | 7.3 |
|  | *BS00067650_51* | 5D | 526464187 | 4.52E-05 | 11.9 |
|  | *GENE-3006_294* | 5D | 558441139 | 6.64E-05 | 10.0 |
|  | *IAAV4275* | 7D | 463039931 | 1.57E-05 | 9.9 |
|  | *Kukri_rep_c69088_774* | 7D | 463039931 | 2.94E-05 | 9.2 |
| SB | *Kukri_c2121_1936* | 1A | 12304604 | 5.15E-04 | 5.2 |
|  | *Excalibur_c54892_188* | 2A | 41985239 | 2.13E-04 | 6.3 |
|  | *BS00022159_51* | 3A | 541260879 | 3.13E-04 | 5.5 |
|  | *BS00065444_51* | 4A | 618033575 | 5.81E-05 | 7.3 |
|  | *Tdurum_contig48766_257* | 5A | 445191816 | 3.19E-04 | 7.3 |
|  | *Kukri_c51091_323* | 3B | 8811363 | 2.54E-04 | 6.0 |
|  | *wsnp_CAP11_c59_99317* | 3B | 779542018 | 3.52E-04 | 5.6 |
|  | *TA004677-0674* | 7B | 336260096 | 2.12E-04 | 5. 9 |
|  | *BobWhite_c13098_670* | 7B | 489827215 | 4.63E-04 | 5.2 |
|  | *BobWhite_c8890_279* | 7B | 650130699 | 5.17E-04 | 6.5 |
|  | *RAC875_c1858_2693* | 2D | 627926191 | 4.57E-04 | 5.4 |
|  | *RAC875_c2660_1206* | 5D | 245313846 | 3.21E-04 | 5.5 |
|  | *GENE-3006_294* | 5D | 558441139 | 4.59E-04 | 6.9 |
|  | *IAAV4275* | 7D | 463039931 | 2.96E-04 | 5.6 |
| PT | *TA005827-0874* | 2A | 51567656 | 3.35E-04 | 7.1 |
|  | *Tdurum_contig19415_271* | 3A | 148603713 | 2.29E-04 | 7.5 |
|  | *Tdurum_contig18901_188* | 5A | 114397941 | 3.09E-04 | 7.3 |
|  | *IACX1098* | 2B | 58324675 | 4.79E-04 | 6.7 |
|  | *CAP12_rep_c5926_51* | 2B | 75694948 | 1.93E-04 | 7.7 |
|  | *CAP12_rep_c5926_115* | 2B | 75695012 | 3.35E-04 | 7.1 |
|  | *Kukri_c43403_594* | 2B | 76817000 | 3.35E-04 | 7.1 |
|  | *Kukri_c43403_412* | 2B | 76817312 | 3.85E-04 | 6.9 |
|  | *Kukri_c43403_346* | 2B | 76817378 | 3.35E-04 | 7.1 |
|  | *Excalibur_rep_c101660_546* | 2B | 77172619 | 3.35E-04 | 7.1 |
|  | *Tdurum_contig29620_125* | 2B | 77173048 | 3.35E-04 | 7.1 |
|  | *Tdurum_contig11350_827* | 2B | 77902878 | 3.35E-04 | 7.1 |
|  | *Tdurum_contig11350_629* | 2B | 77905076 | 3.22E-04 | 7.1 |
|  | *RAC875_c38941_230* | 6B | 117906552 | 4.66E-04 | 7.2 |
|  | *Jagger_c2273_166* | 2D | 49877384 | 3.98E-04 | 6.9 |
| T | *Excalibur_c33675_201* | 5B | 550847442 | 5.88E-05 | 9.1 |
|  | *BS00022437_51* | 6B | 715775218 | 4.45E-04 | 6.8 |

PV, peak viscosity; TV, trough viscosity; BD, breakdown; FV, final viscosity; SB, setback; PT, peak time; T, pasting temperature.

Table S3 Significant single-nucleotide polymorphisms (SNPs) identified by genome-wide association study in multi-environment

| Trait | Multi-  environment | SNP | Chr | Position | R^2^(%) | | | | | | | *P*-value | | | | | | |
| --- | --- | --- | --- | --- | --- | --- | --- | --- | --- | --- | --- | --- | --- | --- | --- | --- | --- | --- |
|  |  |  |  |  | E1 | E2 | E3 | E4 | E5 | E6 | E7 | E1 | E2 | E3 | E4 | E5 | E6 | E7 |
| PV | E2, E7 | *Tdurum_contig56373_348* | 3A | 644622716 |  | 10.7 |  |  |  |  | 11.2 |  | 0.000 |  |  |  |  | 0.000 |
|  | E2, E7 | *BobWhite_c17731_56* | 4A | 689849795 |  | 9.2 |  |  |  |  | 10.9 |  | 0.000 |  |  |  |  | 0.000 |
|  | E2, E7 | *RAC875_c55661_677* | 5A | 52409349 |  | 10.4 |  |  |  |  | 10.6 |  | 0.000 |  |  |  |  | 0.000 |
|  | E2, E7 | *BS00108779_51* | 7A | 139098843 |  | 8.3 |  |  |  |  | 10.8 |  | 0.000 |  |  |  |  | 0.000 |
|  | E2, E7 | *BS00091101_51* | 7A | 142648203 |  | 9.1 |  |  |  |  | 11.6 |  | 0.000 |  |  |  |  | 0.000 |
|  | E2, E7 | *GENE-4428_113* | 7A | 534326357 |  | 11.0 |  |  |  |  | 13.1 |  | 0.000 |  |  |  |  | 0.000 |
|  | E2, E7 | *Ku_c104966_604* | 7A | 534332644 |  | 6.6 |  |  |  |  | 10.5 |  | 0.000 |  |  |  |  | 0.000 |
|  | E2, E7 | *IAAV447* | 7A | 536516307 |  | 6.3 |  |  |  |  | 10.1 |  | 0.000 |  |  |  |  | 0.000 |
|  | E2, E7 | *wsnp_CAP12_rep_c4678_2134259* | 2B | 146634773 |  | 8.9 |  |  |  |  | 12.5 |  | 0.000 |  |  |  |  | 0.000 |
|  | E2, E7 | *BS00078506_51* | 2B | 210842786 |  | 13.5 |  |  |  |  | 13.7 |  | 0.000 |  |  |  |  | 0.000 |
|  | E2, E7 | *Tdurum_contig46867_799* | 4B | 7038188 |  | 11.2 |  |  |  |  | 8.8 |  | 0.000 |  |  |  |  | 0.000 |
|  | E2, E7 | *Ku_c2735_2959* | 5B | 695992955 |  | 8.5 |  |  |  |  | 9.7 |  | 0.000 |  |  |  |  | 0.001 |
|  | E2, E7 | *BobWhite_c13098_670* | 7B | 489827215 |  | 7.7 |  |  |  |  | 11.2 |  | 0.000 |  |  |  |  | 0.000 |
|  | E2, E7 | *IAAV4275* | 7D | 463039931 |  | 12.4 |  |  |  |  | 13.7 |  | 0.000 |  |  |  |  | 0.000 |
|  | E2, E7 | *Kukri_rep_c69088_774* | 7D | 463039931 |  | 11.9 |  |  |  |  | 13.4 |  | 0.000 |  |  |  |  | 0.000 |
|  | E3, E7 | *Ku_c23179_1250* | 3B | 487540433 |  |  | 9.1 |  |  |  | 6.0 |  |  | 0.001 |  |  |  | 0.000 |
|  | E4, E7 | *Excalibur_c9183_1397* | 7D | 13011013 |  |  |  | 10.8 |  |  | 6.2 |  |  |  | 0.000 |  |  | 0.000 |
| TV | E2, E7 | *GENE-4428_113* | 7A | 534326357 |  | 8.4 |  |  |  |  | 7.7 |  | 0.000 |  |  |  |  | 0.000 |
|  | E2, E7 | *Kukri_rep_c69478_1058* | 7A | 536509639 |  | 7.9 |  |  |  |  | 7.4 |  | 0.000 |  |  |  |  | 0.000 |
|  | E2, E7 | *Kukri_c55909_1109* | 2B | 770681399 |  | 8.0 |  |  |  |  | 7.6 |  | 0.000 |  |  |  |  | 0.000 |
|  | E2, E7 | *Kukri_c11040_787* | 2B | 770684873 |  | 7.4 |  |  |  |  | 7.3 |  | 0.000 |  |  |  |  | 0.000 |
|  | E2, E7 | *Tdurum_contig70554_1004* | 5B | 604140767 |  | 6.3 |  |  |  |  | 7.9 |  | 0.000 |  |  |  |  | 0.000 |
|  | E2, E7 | *BobWhite_c13098_670* | 7B | 489827215 |  | 7.3 |  |  |  |  | 6.8 |  | 0.000 |  |  |  |  | 0.000 |
|  | E2, E7 | *IAAV4275* | 7D | 463039931 |  | 9.8 |  |  |  |  | 8.2 |  | 0.000 |  |  |  |  | 0.000 |
|  | E2, E7 | *Kukri_rep_c69088_774* | 7D | 463039931 |  | 9.1 |  |  |  |  | 7.7 |  | 0.000 |  |  |  |  | 0.000 |
| BD | E4, E5, E7 | *Excalibur_c9183_1397* | 7D | 13011013 |  |  |  | 14.0 | 13.3 |  | 7.8 |  |  |  | 0.000 | 0.000 |  | 0.000 |
|  | E1, E3 | *BS00023017_51* | 3B | 779366910 | 10.3 |  |  | 9.6 |  |  |  | 0.000 |  |  | 0.000 |  |  |  |
|  | E3, E6 | *GENE-4993_69* | 7B | 102235375 |  |  | 8.9 |  |  | 11.7 |  |  |  | 0.001 |  |  | 0.000 |  |
|  | E4, E5 | *Tdurum_contig69003_459* | 7D | 13014954 |  |  |  | 11.1 | 13.3 |  |  |  |  |  | 0.000 | 0.000 |  |  |
| FV | E2, E7 | *BS00067650_51* | 5D | 526464187 |  | 13.1 |  |  |  |  | 11.8 |  | 0.000 |  |  |  |  | 0.000 |
| SB | E3, E7 | *Tdurum_contig48766_257* | 5A | 445191816 |  |  | 8.2 |  |  |  | 7.3 |  |  | 0.001 |  |  |  | 0.000 |
| PT | E1, E5 | *IACX6089* | 2A | 15612672 | 9.2 |  |  |  | 11.1 |  |  | 0.001 |  |  |  | 0.000 |  |  |
| T | E2, E4, E5, E7 | *Excalibur_c33675_201* | 5B | 550847442 |  | 8.3 |  | 8.9 | 11.5 |  | 9.1 |  | 0.000 |  | 0.001 | 0.000 |  | 0.000 |
|  | E3, E6, E7 | *BS00022437_51* | 6B | 715775218 |  |  | 10.1 |  |  | 11.6 | 6.7 |  |  | 0.000 |  |  | 0.000 | 0.000 |
|  | E1, E2, E5 | *wsnp_Ex_c10251_16815792* | 2B | 608201876 | 11.8 | 7.5 |  |  | 9.5 |  |  | 0.000 | 0.000 |  |  | 0.000 |  |  |
|  | E3, E4 | *Excalibur_c10124_361* | 7B | 446178433 |  |  | 18.2 | 9.9 |  |  |  |  |  | 0.000 | 0.000 |  |  |  |
|  | E3, E4 | *Kukri_c4560_110* | 7B | 450680091 |  |  | 18.2 | 9.9 |  |  |  |  |  | 0.000 | 0.000 |  |  |  |
|  | E3, E4, E6 | *Excalibur_rep_c67475_1759* | 7B | 498523612 |  |  | 14.8 | 14.3 |  | 9.2 |  |  |  | 0.000 | 0.000 |  | 0.000 |  |
|  | E3, E5 | *Excalibur_c34849_295* | 6D | 270567915 |  |  | 12.2 |  | 9.5 |  |  |  |  | 0.000 | ` | 0.000 |  |  |
|  | E4, E6 | *RAC875_c49999_66* | 2B | 89557717 |  |  |  | 9.4 |  | 10.1 |  |  |  |  | 0.000 |  | 0.000 |  |
|  | E4, E5, E6 | *RAC875_c7610_81* | 7B | 457788083 |  |  |  | 8.8 | 10.3 | 10.2 |  |  |  |  | 0.001 | 0.000 | 0.000 |  |

E1, 2012_ES; E2, 2012_SHZ; E3, 2012_ZS; E4, 2013_ES; E5, 2013_SHZ; E6, 2013_ZS, which represent 2012 and 2013 cropping seasons in Er’shi (ES), Shihezi (SHZ) and Zhaosu (ZS), respectively; E7, BLUP, best linear unbiased predictor of RVA parameters in 192 wheat cultivars during two cropping seasons across three environments; Chr, chromosome.

Table S4 Candidate genes identified by genome-wide association study

| Trait | SNP name | Candidate gene | Annotation | Protein name |
| --- | --- | --- | --- | --- |
| PV | *Tdurum_contig56373_348* | *TraesCS3A02G396900* | 5'UTR | —— |
|  | *BobWhite_c17731_56* | *TraesCS4A02G419400* | 3'UTR | Arginine decarboxylase, EC 4.1.1.19 |
|  | *RAC875_c55661_677* | *——* | intergenic | —— |
|  | *BS00108779_51* | *——* | intergenic | —— |
|  | *BS00091101_51* | *TraesCS7A02G187100* | CDS | DCD domain-containing protein |
|  | *GENE-4428_113* | *TraesCS7A02G360700* | intron | Phospholipid-transporting ATPase, EC 7.6.2.1 |
|  | *Ku_c104966_604* | *TraesCS7A02G360800* | CDS | —— |
|  | *IAAV447* | *TraesCS7A02G362000* | 3’UTR | Transmembrane 9 superfamily member |
|  | *wsnp_CAP12_rep_c4678_2134259* | *TraesCS2B02G172100* | 3'UTR | Xylulose kinase, EC 2.7.1.17 |
|  | *BS00078506_51* | *TraesCS2B02G220600* | intron | AB hydrolase-1 domain-containing protein |
|  | *Tdurum_contig46867_799* | *TraesCS4B02G010400* | CDS | Aldo_ket_red domain-containing protein |
|  | *Ku_c2735_2959* | *TraesCS5B02G541500* | CDS | —— |
|  | *BobWhite_c13098_670* | *TraesCS7B02G266800* | CDS | —— |
|  | *IAAV4275* | *TraesCS7D02G360200* | CDS | —— |
|  | *Kukri_rep_c69088_774* | *TraesCS7D02G360200* | CDS | —— |
|  | *Ku_c23179_1250* | *TraesCS3B02G303400* | CDS | —— |
|  | *Excalibur_c9183_1397* | *TraesCS7D02G026700* | CDS | 1,3-beta-glucan synthase, EC 2.4.1.34 |
| TV | *GENE-4428_113* | *TraesCS7A02G360700* | intron | Phospholipid-transporting ATPase, EC 7.6.2.1 |
|  | *Kukri_rep_c69478_1058* | *——* | intergenic | —— |
|  | *Kukri_c55909_1109* | *TraesCS2B02G583300* | CDS | Vacuolar protein sorting-associated protein 41 homolog |
|  | *Kukri_c11040_787* | *TraesCS2B02G583300* | CDS | Vacuolar protein sorting-associated protein 41 homolog |
|  | *Tdurum_contig70554_1004* | *TraesCS5B02G428500* | CDS | —— |
|  | *BobWhite_c13098_670* | *TraesCS7B02G266800* | CDS | —— |
|  | *IAAV4275* | *TraesCS7D02G360200* | CDS | —— |
|  | *Kukri_rep_c69088_774* | *TraesCS7D02G360200* | CDS | —— |
| BD | *Excalibur_c9183_1397* | *TraesCS7D02G026700* | CDS | 1,3-beta-glucan synthase, EC 2.4.1.34 |
|  | *BS00023017_51* | *TraesCS3B02G540400* | CDS | CaM_binding domain-containing protein |
|  | *GENE-4993_69* | *TraesCS7B02G087400* | intron | —— |
|  | *Tdurum_contig69003_459* | *TraesCS7D02G026700* | CDS | 1,3-beta-glucan synthase, EC 2.4.1.34 |
| FV | *BS00067650_51* | *TraesCS5D02G494400* | intron | Protein kinase domain-containing protein |
| SB | *Tdurum_contig48766_257* | *TraesCS5A02G229400* | 3'UTR | Peptidyl-prolyl cis-trans isomerase |
| PT | *IACX6089* | *TraesCS2A02G036300* | CDS | —— |
| T | *Excalibur_c33675_201* | *TraesCS5B02G373000* | CDS | RING-type E3 ubiquitin transferase |
|  | *BS00022437_51* | *——* | intergenic | —— |
|  | *wsnp_Ex_c10251_16815792* | *——* | intergenic | —— |
|  | *Excalibur_c10124_361* | *TraesCS7B02G239800* | CDS | —— |
|  | *Kukri_c4560_110* | *——* | intergenic | —— |
|  | *Excalibur_rep_c67475_1759* | *TraesCS7B02G271600* | CDS | —— |
|  | *Excalibur_c34849_295* | *——* | intergenic | —— |
|  | *RAC875_c49999_66* | *TraesCS2B02G122400* | CDS | PA domain-containing protein |
|  | *RAC875_c7610_81* | *TraesCS7B02G247600* | CDS | zinc_ribbon_12 domain-containing protein |

PV, peak viscosity; TV, trough viscosity; BD, breakdown; FV, final viscosity; SB, setback; PT, peak time; T, pasting temperature.

Table S5 *P*-values of *t*-tests for efficacy of different alleles on RVA traits

| Trait | SNP name | Allele | | Number | | *P*-value | | | | | |
| --- | --- | --- | --- | --- | --- | --- | --- | --- | --- | --- | --- |
|  |  |  |  |  |  | 2012_ES | 2012_SHZ | 2012_ZS | 2013_ES | 2013_SHZ | 2013_ZS |
| PV | *Tdurum_contig56373_348* | AA | CC | 137 | 3 | 0.446 | 0.011 | 0.898 | 0.071 | 0.580 | 0.323 |
|  | *BobWhite_c17731_56* | CC | TT | 5 | 134 | 0.043 | 0.009 | 0.158 | 0.484 | 0.875 | 0.286 |
|  | *RAC875_c55661_677* | CC | TT | 185 | 4 | —— | 0.071 | —— | —— | —— | —— |
|  | *BS00108779_51* | CC | TT | 7 | 132 | 0.415 | 0.664 | 0.581 | 0.720 | 0.286 | 0.900 |
|  | *BS00091101_51* | AA | GG | 133 | 6 | 0.115 | 0.708 | 0.879 | 0.477 | 0.334 | 0.735 |
|  | *GENE-4428_113* | CC | TT | 5 | 134 | 0.439 | 0.000 | 0.101 | 0.026 | 0.087 | 0.074 |
|  | *Ku_c104966_604* | AA | GG | 135 | 5 | 0.536 | 0.000 | 0.355 | 0.200 | 0.181 | 0.159 |
|  | *IAAV447* | AA | GG | 5 | 135 | 0.536 | 0.000 | 0.355 | 0.200 | 0.181 | 0.159 |
|  | *wsnp_CAP12_rep_c4678_2134259* | AA | GG | 137 | 2 | 0.660 | 0.000 | 0.019 | 0.553 | 0.474 | 0.242 |
|  | *BS00078506_51* | CC | TT | 3 | 135 | 0.707 | 0.004 | 0.126 | 0.552 | 0.531 | 0.898 |
|  | *Tdurum_contig46867_799* | GG | TT | 134 | 5 | 0.357 | 0.000 | 0.908 | 0.710 | 0.279 | 0.085 |
|  | *Ku_c2735_2959* | AA | CC | 35 | 99 | 0.668 | 0.206 | 0.698 | 0.030 | 0.459 | 0.015 |
|  | *BobWhite_c13098_670* | CC | TT | 135 | 4 | 0.105 | 0.000 | 0.389 | 0.067 | 0.243 | 0.131 |
|  | *IAAV4275* | CC | TT | 4 | 134 | 0.600 | 0.000 | 0.095 | 0.020 | 0.113 | 0.098 |
|  | *Kukri_rep_c69088_774* | CC | TT | 4 | 134 | —— | 0.000 | 0.095 | 0.020 | 0.113 | 0.098 |
|  | *Ku_c23179_1250* | CC | TT | 29 | 110 | 0.097 | 0.000 | 0.809 | 0.289 | 0.028 | 0.328 |
|  | *Excalibur_c9183_1397* | CC | TT | 31 | 106 | 0.819 | 0.767 | 0.157 | 0.798 | 0.789 | 0.213 |
| TV | *GENE-4428_113* | CC | TT | 5 | 134 | 0.870 | 0.000 | 0.828 | 0.290 | 0.889 | 0.238 |
|  | *Kukri_rep_c69478_1058* | AA | GG | 134 | 5 | 0.870 | 0.000 | 0.828 | 0.290 | 0.889 | 0.238 |
|  | *Kukri_c55909_1109* | AA | GG | 122 | 17 | 0.486 | 0.305 | 0.394 | 0.011 | 0.684 | 0.932 |
|  | *Kukri_c11040_787* | AA | GG | 122 | 16 | 0.805 | 0.192 | 0.492 | 0.012 | 0.681 | 0.926 |
|  | *Tdurum_contig70554_1004* | GG | TT | 4 | 136 | 0.285 | 0.000 | 0.082 | 0.019 | 0.787 | 0.576 |
|  | *BobWhite_c13098_670* | CC | TT | 135 | 4 | 0.530 | 0.000 | 0.249 | 0.252 | 0.863 | 0.316 |
|  | *IAAV4275* | CC | TT | 4 | 134 | 0.703 | 0.000 | 0.632 | 0.096 | 0.600 | 0.146 |
|  | *Kukri_rep_c69088_774* | CC | TT | 4 | 134 | 0.703 | 0.000 | 0.632 | 0.096 | 0.600 | 0.146 |
| BD | *Excalibur_c9183_1397* | CC | TT | 30 | 107 | 0.548 | 0.878 | 0.590 | 0.974 | 0.599 | 0.129 |
|  | *BS00023017_51* | AA | AC | 83 | 54 | 0.379 | 0.011 | 0.259 | 0.015 | 0.042 | 0.027 |
|  | *GENE-4993_69* | GG | TT | 10 | 127 | 0.361 | 0.000 | 0.644 | 0.245 | 0.314 | 0.400 |
|  | *Tdurum_contig69003_459* | CC | TT | 110 | 30 | 0.547 | 0.821 | 0.495 | 0.991 | 0.601 | 0.130 |
| FV | *BS00067650_51* | CC | TT | 121 | 16 | 0.975 | 0.009 | 0.965 | 0.309 | 0.589 | 0.232 |
| SB | *Tdurum_contig48766_257* | AA | GG | 114 | 25 | 0.225 | 0.909 | 0.367 | 0.383 | 0.047 | 0.068 |
| PT | *IACX6089* | CC | TT | 8 | 120 | 0.937 | 0.274 | 0.423 | 0.074 | 0.227 | 0.446 |
| T | *Excalibur_c33675_201* | CC | TT | 100 | 32 | 0.917 | 0.024 | 0.094 | 0.441 | 0.035 | 0.102 |
|  | *BS00022437_51* | CC | NC | 110 | 24 | 0.784 | 0.239 | 0.287 | 0.350 | 0.213 | 0.256 |
|  | *wsnp_Ex_c10251_16815792* | AA | GG | 123 | 8 | 0.710 | 0.002 | 0.997 | 0.597 | 0.170 | 0.850 |
|  | *Excalibur_c10124_361* | CC | TT | 107 | 27 | 0.005 | 0.024 | 0.010 | 0.006 | 0.021 | 0.002 |
|  | *Kukri_c4560_110* | CC | TT | 107 | 27 | 0.005 | 0.523 | 0.010 | 0.006 | 0.021 | 0.002 |
|  | *Excalibur_rep_c67475_1759* | CC | TT | 27 | 106 | 0.113 | 0.431 | 0.040 | 0.026 | 0.108 | 0.009 |
|  | *Excalibur_c34849_295* | CC | NC | 131 | 3 | 0.467 | 0.182 | 0.016 | 0.024 | 0.340 | —— |
|  | *RAC875_c49999_66* | CC | NC | 119 | 15 | 0.000 | 0.583 | 0.000 | 0.000 | 0.000 | 0.000 |
|  | *RAC875_c7610_81* | AA | GG | 73 | 60 | 0.417 | 0.348 | 0.337 | 0.884 | 0.953 | 0.616 |

PV, peak viscosity; TV, trough viscosity; BD, breakdown; FV, final viscosity; SB, setback; PT, peak time; T, pasting temperature. 2012_ES, 2012_SHZ, 2012_ZS, 2013_ES, 2013_SHZ and 2013_ZS represent 2012 and 2013 cropping seasons in Er’shi (ES), Shihezi (SHZ) and Zhaosu (ZS), respectively.

Table S6 Haplotypes with different alleles in the blocks

| Trait | Chr | Interval | Block | SNP | Haplotype | Number of cultivars |  |
| --- | --- | --- | --- | --- | --- | --- | --- |
|  |  |  |  |  |  |  |  |
| T | 7B | 442-454 Mb | Block1 | *CAP12_c8025_110/IAAV2037/Excalibur_c49622_60* | CC/AA/CC | 2 |  |
|  |  |  | Block2 | *wsnp_Ex_c64815_63464750/RAC875_c4438_419/IAAV3414/TA001679-0356* | AA/TT/GG/GG | 17 |  |

T, pasting temperature; Chr, chromosome.


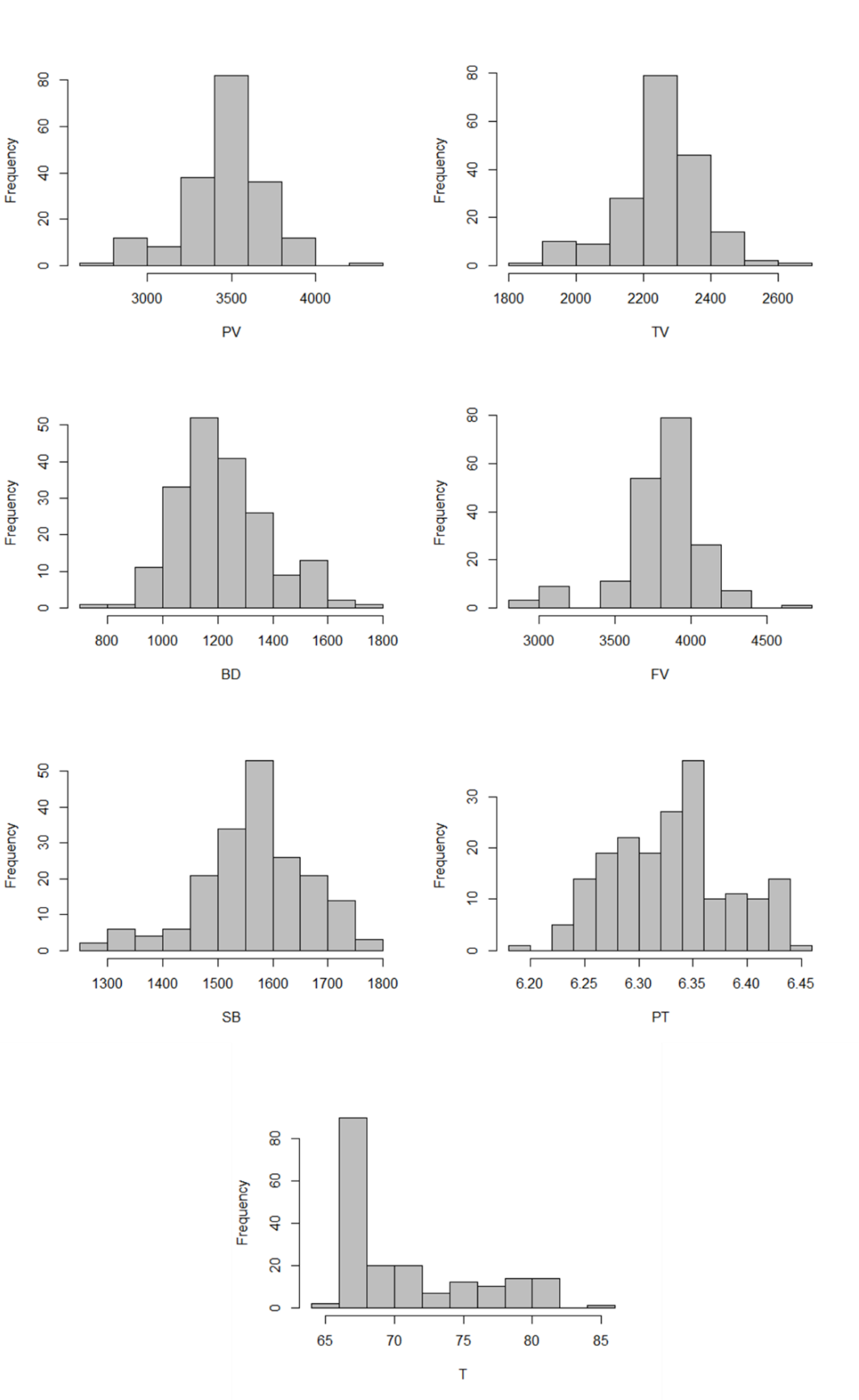


Fig. S1 Frequency distributions of BLUP values of RVA parameters in 192 Chinese spring wheat cultivars. PV, peak viscosity; TV, trough viscosity; BD, breakdown; FV, final viscosity; SB, setback; PT, peak time; T, pasting temperature.


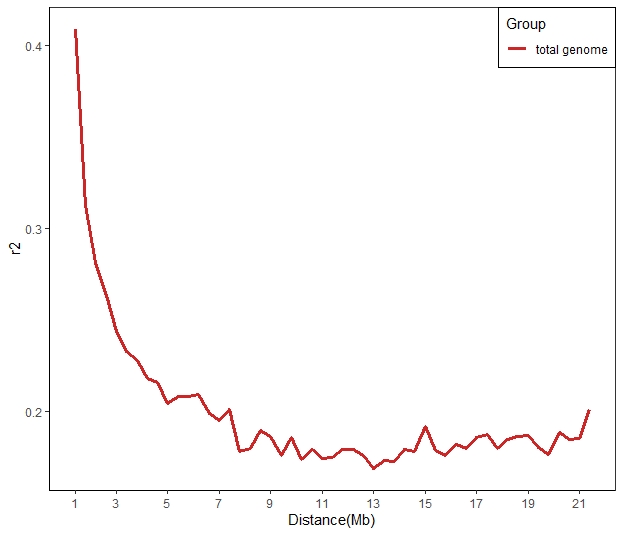


**Fig. S2** Decay of linkage disequilibrium (LD) in the total genome of the Chinese spring wheat.


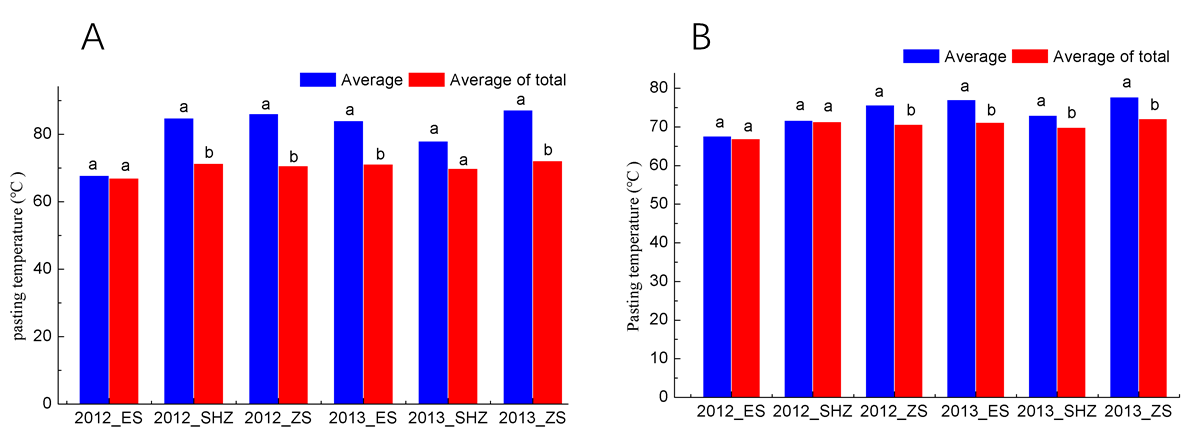


**Fig. S3** Phenotypic effects caused by haplotypes in different environments. **(A)** Block 1. **(B)** Block 2. 2012_ES, 2012_SHZ, 2012_ZS, 2013_ES, 2013_SHZ and 2013_ZS represent 2012 and 2013 cropping seasons in Er’shi (ES), Shihezi (SHZ) and Zhaosu (ZS), respectively. Different lowercase letters indicate significant differences at *P* < 0.05.
